# NUTRITIONAL ASPECTS AND THE USE OF NUTRITIONAL SUPPLEMENTS BY WOMEN WHO UNDERWENT GASTRIC BYPASS

**DOI:** 10.1590/0102-6720201700010004

**Published:** 2017

**Authors:** Elisangela Mara TRINDADE, Telma Souza e Silva GEBARA, Maria Paula Carlini CAMBI, Giorgio Alfredo Pedroso BARETTA

**Affiliations:** Clínica Baretta, Curitiba, PR, Brazil.

**Keywords:** Gastric bypass, Nutritional deficiencies, Nutritional supplements.

## Abstract

**Background:**

Bariatric surgery is deemed one of the most effective procedures for the treatment of obesity and it aims at the reduction and maintenance of weight loss in long term, as the control of the related comorbidities.

**Aim:**

Quantify the occurrence of alterations of the gastrointestinal tract, suggestive signs of nutritional deficiencies and the use of supplements in a group of women undergoing bariatric surgery.

**Methods:**

The sample consisted of women aged 20-65 years submitted to Roux-en-Y gastric bypass with monitoring equal to or higher than 24 months. For the qualitative analysis, the Feeding Frequency Questionnaire was used.

**Results:**

In the postoperative period, alopecia was the most reported (79.3%), followed by changes in the texture of the nails, both considered predictive of nutritional deficiencies. Changes in the gastrointestinal tract were described in 86.2%, and episodes of dumping were reported in 65.5%. Qualitative analysis has shown reduced daily consumption of sources of animal and plant proteins.

**Conclusion:**

After bariatric surgery can occur flatulence, vomiting and dumping syndrome as the most frequent representative symptoms of digestive functional disorders. Alopecia and nail changes are the most important signs of nutritional deficiency. The use of dietary supplements in the postoperative period is scarce and sporadic.

## RESUMO

**Racional:**

A cirurgia bariátrica é considerada um dos procedimentos mais eficazes para tratamento da obesidade e objetiva a redução e manutenção da perda de peso em longo prazo, assim como, o controle das comorbidades associadas.

**Objetivo:**

Quantificar a ocorrência de alterações funcionais do trato gastrointestinal, sinais sugestivos de carências nutricionais e o uso de suplementos em um grupo de mulheres submetidas à cirurgia bariátrica.

**Métodos:**

A amostra foi constituída por mulheres com idades entre 20-65 anos submetidas ao bypass gástrico em Y-de-Roux com seguimento igual ou superior a 24 meses. Para análise qualitativa foi utilizado o Questionário de Frequência Alimentar.

**Resultados:**

No período pós-operatório a alopecia foi a mais relatada (79,3%), seguida de alteração na textura das unhas, ambas consideradas preditivos de carências nutricionais. As alterações do trato gastrointestinal foram descritas por 86,2%, e os episódios de dumping foram relatados em 65,5%. Na análise qualitativa foi observado consumo diário reduzido de fontes de proteínas animal e vegetal.

**Conclusão:**

Após a cirurgia bariátrica pode ocorrer flatulência, vômitos e síndrome de dumping como sintomas representativos mais frequentes das alterações funcionais digestivas. Alopecia e alterações ungueais são os sinais mais relevantes de carência nutricional. O uso de suplementos alimentares no pós-operatório é escasso e esporádico.

## INTRODUCTION

Bariatric surgery provides greater reduction in weight, being also responsible for the control and even the removal of comorbidities associated with obesity^9^ ^,^ ^10^. However, further dietary restrictions may trigger nutritional deficiencies, including anemia, bone mass loss, protein malnutrition, peripheral neuropathies, poor visual acuity, Wernicke's encephalopathy and even impaired fetal growth^1^ ^,^ ^7^ ^,^ ^14^.

When undiagnosed, common occurrence, changes and nutritional deficiencies will certainly compromise the quality of life. Thus, the nutritional monitoring is important to avoid intolerances, nutritional deficiencies due to poor feeding and excessive loss of weight^13^.

The aim of this study was to quantify the occurrence of functional gastrointestinal tract changes, suggestive signs of nutritional deficiencies and the use of supplements in a group of women undergoing bariatric surgery.

## METHODS

This study was approved by the Ethics and Research Committee of the Sociedade Evangélica Beneficente de Curitiba, PR, according to the resolution 466/2012 of the National Commission for Research Ethics 3, with the consolidated expert report 1199826. It is a prospective cross-sectional study held at Clínica Baretta, Curitiba, PR, Brazil. Data collection was conducted in September and October 2015.

The sample consisted of women aged 20-65 years undergoing surgical treatment for obesity with Fobi-Capella technique and the postoperative period equal to or higher than 24 months.

The following were considered as exclusion criteria: individuals who had surgical complications in the immediate postoperative period, pregnancy, cancer, and those who agreed to participate in the survey or had difficulty answering the questionnaire, in addition to withdrawal at any time. Participants read, agreed and signed the Informed Consent Form.

Data collection was performed from a self-administered questionnaire, adapted from Viana, Cardoso^19^, composed of closed questions that were answered without interference from researchers and a Feeding Frequency Questionnaire (FFQ) adapted from Fisberg et al.^8^. Both questionnaires were made available in physical (printed) and electronic formats, the electronic was sent via email for the individuals registered in the service, and the printed copies are given and explained to those attending to the clinic during the period of data collection.

### Statistical analysis

The results were statistically treated with the support of R Software, R Development Core Team^15^ language version 3.2.2. Normality tests, T-paired test or Wilcoxon were used for the situations before and after, contingency tables and the accurate Fisher test to measure the association in counting data. 

## RESULTS

The alopecia was the suggestive clinical change of nutritional deficiencies most mentioned and reported by 79.3% (n=23) of the participants, followed by change in the texture of the nails by 62.1% (n=18) and in the skin by 24.1% (n=7). 

Changes and symptoms related to the functioning of the gastrointestinal tract after surgery were described by 86.2% (n=25) of the interviewes. Flatulence and vomiting had an incidence in 44.8% (n=13) and 41.4% (n=12) respectively. On the other hand, the dumping syndrome was reported by 65.5% (n=19).

The Table 1 contains the quantitative analysis of feeding frequency from the application of the QFA^8^. 

**TABLE 1:** Frequency of food consumption of survey participants

Food	Frequency of patients feeding											
	Daily		Weekly		Monthly		Rare		Do not consume		Total	
	n	%	n	%	n	%	n	%	n	%	n	%
Fruits	16	55,2	9	31,0	1	3,4	3	10,3	0	0,0	29	100,0
Vegetables	21	72,4	6	20,7	1	3,4	1	3,4	0	0,0	29	100,0
Beans	11	37,9	13	44,8	2	6,9	3	10,3	0	0,0	29	100,0
Meat and eggs	22	75,9	4	13,8	0	0,0	3	10,3	0	0,0	29	100,0
Milk and dairy	25	86,2	1	3,4	0	0,0	2	6,9	1	3,4	29	100,0

n=number of participants; %=percentage of participants

The rare consumption of beans was mentioned by 10.3% (n=3). Regarding the meat and eggs group, 75.9% (n=22) reported daily consumption and 10.3% (n=3) eventual or rare. 

**FIGURE 1:**
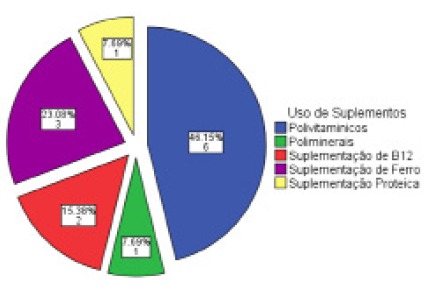
Use of supplements during the postoperative period of bariatric surgery

The use of nutritional supplements was alleged by 31% (n=9) of the survey participants and denied by 69% (n=20). Among those using nutritional supplements, 44.4% (n=4) reported their use during the period of 12-24 months. The main supplements used were proteins, pluriminerals, plurivitamins iron and vitamin B12 (Figure 1).

## DISCUSSION

The dumping syndrome is characterized by a number of symptoms presented soon after the food intake of carbohydrates and fats, resulting from accelerated gastric emptying in patients undergoing bariatric surgery^16^. According to the comparative study of Faria et al.^6^, regarding the Roux-en-Y gastric bypass with and without placing the containment ring, the use of gastric containment ring reduces the consumption of protein and fiber, favoring a lower incidence of vomiting. In the study of Silva e Gomes^17^ there was found that the interposition of jejunal loop contributes to reduction in episodes of dumping syndrome in individuals that underwent gastrectomy.

Analysis of the daily food intake was carried out after the classification of food groups compared with the recommendations proposed by the food pyramid adapted from Moizé et al.^4^ ^,^ ^11^ ^,^ ^12^. However, for being a qualitative analysis of the QFA, the number of daily servings of each group were not considered. According to this analysis, there was a reduction in the consumption group of meat and beans. As seen in Soares, Falcão^18,^ the intake reduction of the meat group may be related to the difficulty of digestion, arising out from reducing gastric capacity and limited availability of pepsin and hydrochloric acid, which promotes gastric discomfort after ingestion. On the other hand, the reduction in beans consumption may be given to the incidence of flatulencies arising from the intake of oligosaccharides.

Nutritional deficiencies are more common in techniques that promote malabsorption due to physiological changes that they produce. The most commonly found ones are related to the proteins, folate, vitamin B_12_, iron, zinc, calcium and vitamin D and occur mostly between 12-15 months of postoperative period. The hypovitaminosis D may occur earlier and the supplementation is recommended, since it is associated with absorption of calcium^5^.

The women gathered in this study reported less adherence to the use of vitamin and mineral supplements, with consequent complaints compatible with alopecia, brittle nails, fatigue, difficulty concentrating, and iron deficiency and megaloblastic anemia. Intestinal malabsorption in gastric bypass can cause serious deficiencies of hydro and liposoluble vitamins, proteins and minerals like iron, calcium, zinc and magnesium. It is frequently verified, in an empirical manner, that the operated person has a nutritional deficits and a greater difficulty in losing and maintaining weight in the long term.

Regular use of the nutritional supplement has been advocated when used correctly, that is, at least five times a week; however, in general, less than half of patients follow this recommendation. The use of plurivitamins/minerals preventively should compose the care protocol for all patients undergoing bariatric surgery, especially those with techniques that involve some degree of malabsorption^2^.

Patients undergoing disabsorptive surgical procedures should use plurivitamins and minerals preventively. The treatment of nutritional deficiencies should consist of megadosage of micronutrients, taking into account the decreased bioavailability, lower absorption and intake area resulting from surgical technique^5^.

Most participants deny the use of dietary supplements in the postoperative period, and among those who did, a representative number reported their use during the period of 12-24 months. The main supplements used were proteins, multiminerals, multivitamins, iron and vitamin B12.

Postoperative nutritional monitoring should be performed with special attention of the nutritionist, since dietary restriction imposed by surgical procedures can result in poor intake of energy and nutrients. Thus, the process of effective weight loss and maintenance requires the quality of food and nutritional supplementations.

## CONCLUSION

After bariatric surgery, one may have flatulence, vomiting and dumping syndrome as the most frequent representative symptoms of digestive functional disorders. Alopecia and nail changes are the most important signs of nutritional deficiency. The use of dietary supplements in the postoperative period is scarce and sporadic. 
